# Substrate-based ablation of atypical atrial flutter in patients with atrial cardiomyopathy

**DOI:** 10.1016/j.ijcha.2022.101018

**Published:** 2022-04-18

**Authors:** Alexander Pott, Yannick Teumer, Karolina Weinmann, Michael Baumhardt, Christiane Schweizer, Dominik Buckert, Carlo Bothner, Wolfgang Rottbauer, Tillman Dahme

**Affiliations:** Department of Medicine II, Ulm University Medical Center, Ulm, Germany

**Keywords:** Atypical flutter, Catheter ablation, Substrate, Anatomical lines, Atrial cardiomyopathy

## Abstract

**Background:**

Standard therapy of atypical atrial flutter (AFL) aims at deploying ablation lines between two non-conducting anatomical structures, thereby creating a line of block within the re-entry circuit. We have developed an ablation strategy, where we incorporate voltage information as a surrogate for atrial fibrosis from the electro-anatomical map (EAM) during AFL ablation procedures to create individualized, substrate-based ablation lines along the area of most pronounced low-voltage within the reentry-circuit.

**Objective:**

The aim of this study was to evaluate acute procedural success and long-term outcome of a substrate-based ablation (SBA) strategy in comparison to a standard anatomically based ablation (ABA) strategy for the ablation of atypical AFL.

**Methods:**

Patients that underwent ablation for AFL at our institution were included. SBA procedures were compared to ABA procedures. Endpoints were acute termination of AFL and recurrence of the index AFL or any other AFL during follow-up.

**Results:**

We included 47 patients, 24 individuals (51.1%) in the SBA group and 23 patients (48.9%) in the ABA group. Most patients had signs of atrial cardiomyopathy, namely enlarged left atrial diameter (LAD) and extended amount of left atrial low-voltage areas (LVA). Termination of AFL occurred in 27 of 29 (93.1%) AFL in the SBA group and in 28 of 31 (90.3%) AFL in the ABA group (p = 0.99). Freedom from recurrence of any atypical AFL after 2.5 years was 21.5% in the ABA group compared to 48.8% in the SBA group (p = 0.047).

**Conclusion:**

Substrate-based ablation is as effective as an anatomically-based ablation in the acute termination of AFL but yields better rhythm outcome with less recurrence of AFL in patients with atrial cardiomyopathy.

## 1 Introduction

Atrial flutter describes atrial macro-reentrant tachycardias. Atypical atrial flutter (AFL) comprises macro-re-entrant tachycardias of the left and/or right atrium, the majority occurring in the left atrium.

AFL typically occurs in elderly patients with previous cardiac surgery, atrial ablation procedures, mitral valve defects or structural heart disease. Since catheter ablation for the treatment of atrial fibrillation (AF) has become standard of care also in aging patients with the number of procedures constantly rising, incidence of AFL is continuously increasing [1], [2], [3].

In contrast to typical atrial flutter, which can be treated with high success rates by ablation of an anatomically well-defined structure, the cavotricuspid isthmus (CTI), identification of the AFL defining re-entry mechanism is much more challenging. The central non-conductive anatomical structure, the center of the macro-reentry in AFL, is typically the mitral valve (MV) or the septal or lateral pulmonary veins (PV), resulting in perimitral, roof-dependent around the lateral PV and roof-dependent around the septal PV as the most common reentrant circuits in left atrial AFL.

The common approach to treat AFL is the 3D-mapping system guided identification of the macro-reentrant circuit and introduction of an ablation line that connects the central anatomical structure with a second non-conducting anatomical structure. A “classical” mitral isthmus line from the left inferior pulmonary vein (LIPV) to the posterior mitral annulus or an anterior mitral isthmus line from the left superior pulmonary vein (LPSV) to the anterior mitral annulus in perimitral AFL or a roof line from the LSPV to the right superior pulmonary vein (RSPV) for roof-dependent AFL is applied, thereby creating a line of block within the reentrant circuit [2], [3], [4], [5]. Establishing a durable bidirectional line of block remains challenging in this anatomically based ablation (ABA) strategy and incomplete transmural ablation lesions often leave proarrhythmogenic substrate behind, thus promoting further atrial tachyarrhythmias [6], [7], [8].

High-Density 3D-Mapping is usually applied to obtain activation maps by gathering local activation time (LAT) during ongoing reentrant tachycardia for ABA strategies. In electroanatomical mapping (EAM) voltage maps are collected simultaneously annotating local electrical signal amplitudes, but these maps are generally not used to determine ablation strategy in AFL ablation cases. ABA strategies for AFL treatment obtain high procedural success rates with reliable termination of most macro-reentrant tachycardias.

However, the introduced line of block does not take low voltage areas into account and thus ablation lines may be introduced in healthy and sometimes thick myocardium possibly impeding durable transmural lesions and leaving substrate for further reentrant circuits behind.

Hence, we hypothesized, that ablation lines incorporating pre-existing left atrial substrate, identified by EAM during tachycardia, might have the potential to address the AFL underlying myocardial substrate allowing a more pathophysiological based ablation approach. However, systematic data on procedural success and outcome of a substrate guided ablation approach in comparison to the conventional ablation strategy for treatment of AFL is sparse and clinical benefit of the ablation strategies targeting for left atrial substrate over the conventional strategy has not been shown yet [9], [10], [11].

**Fig. 1:**
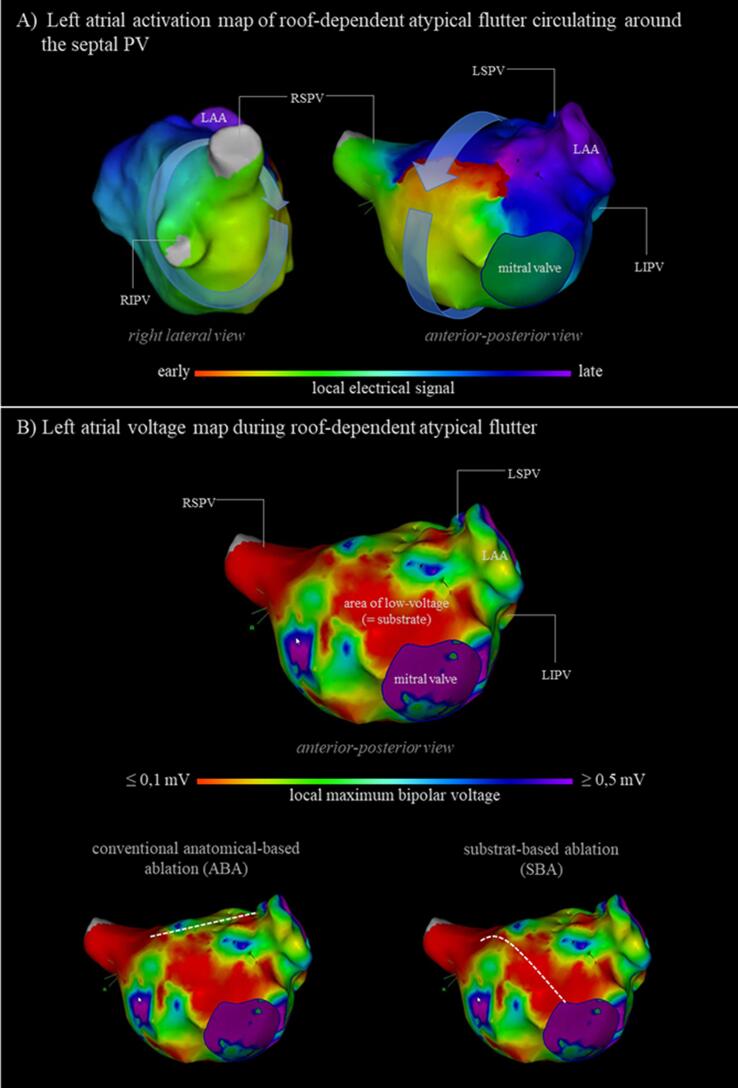
Electro-anatomical Mapping (EAM) of left atrial macro-reentry tachycardia.

In this study, we analysed our ablation strategy, where we incorporate voltage information as a surrogate for atrial fibrosis or scar areas from the EAM during AFL mapping to create individualized, substrate-based ablation (SBA) lines along the area of most pronounced low-voltage within the re-entry circuit (Fig. 1). The aim of our study was to evaluate procedural characteristics and clinical long-term success of our SBA approach in comparison to the conventional ABA approach for the treatment of patients suffering from AFL.

## 2 Methods

### 2.1 Study population

In this single-center study, we included consecutive patients who underwent first time ablation procedure for the treatment of left atrial AFL at our electrophysiology center. To be eligible for inclusion, complete procedural data, especially data set of the 3D mapping procedure and clinical outcome data beyond 6 months after index procedure had to be available. Prior ablation procedures for non-AFL arrhythmia did not lead to patient́s exclusion. The ablation strategy, either SBA or ABA was at the discretion of the operator. Patients were not randomized.

**Supplementary figure 1:**
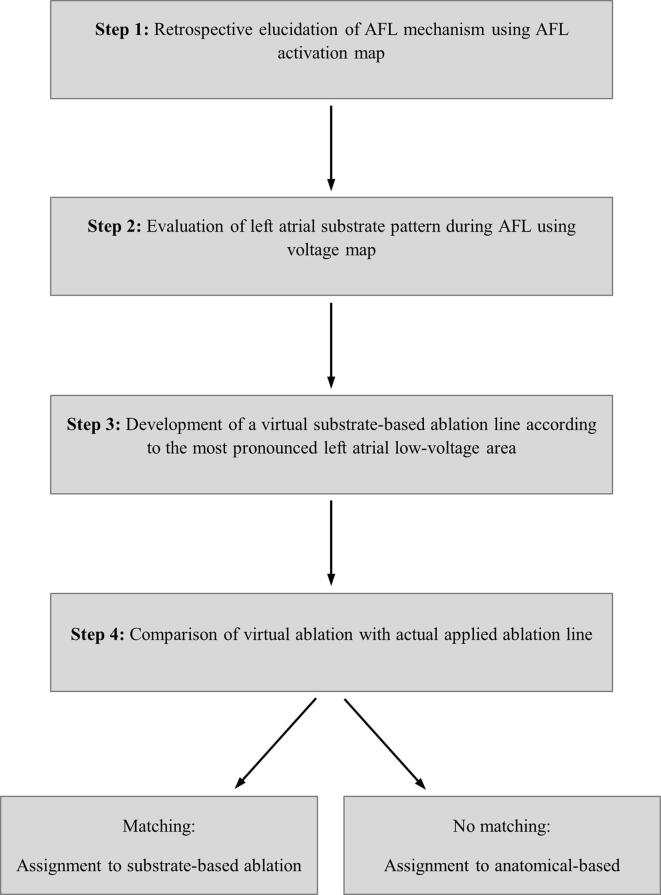
Standardized analysis algorithm by which patients were assigned to either the SBA or the ABA group.

The study population was retrospectively divided into either the SBA or the ABA group according to the applied ablation strategy. Applied ablation strategy was classified in a blinded process, in which three independent electrophysiologists were involved. For this purpose, the 3D mapping data of the index procedure of each patient were anonymized and all references to the selected AFL ablation strategy, especially localization and number of applied ablation points were hidden from the examiner. Supplementary Fig. 1 shows our standardized analysis algorithm, by which patients were assigned to either the SBA or the ABA group.

In a first step, index AFL mechanism had to be elucidated from the corresponding left atrial activation map. In a second step, left atrial substrate pattern was evaluated in the corresponding voltage map. Using both, activation as well as voltage map information, a virtual substrate-based ablation line along the area of most pronounced low-voltage within the re-entry circuit was proposed. Subsequently, procedural characteristics were unblinded and virtual substrate-based ablation line was compared to the actual applied ablation line. If both, the virtual and the actual ablation line were identical, applied ablation strategy was considered as substrate-based ablation (SBA), otherwise ablation was considered as anatomical-based ablation (ABA). All patients gave written informed consent prior to the ablation procedure. The study complies with the Declaration of Helsinki and was approved by the local ethics committee of Ulm University (reference number: 324/16).

### 2.2 Preprocedural management and AFL ablation

Preprocedural management was performed as described before [12], [13]. Briefly, left atrial thrombus was ruled out by transesophageal echocardiography prior ablation. Vitamin K antagonists (VKA) were administered uninterruptedly to a target INR of 2.0 – 2.5 at the time of procedure. Patients treated with non-VKA oral anticoagulants (NOACs) were advised to hold their anticoagulant up to 24 h before the ablation.

In all patients, ablation procedure was performed by using irrigated-tip radiofrequency ablation in combination with a 3D-mapping system (Carto3, Biosense Webster, Irvine CA or NavX Ensite Velocity, St. Jude Medical, St. Paul, MN). The ablation procedure was performed under deep sedation using propofol, midazolam, and/or remifentanil. A 10-polar diagnostic catheter was placed in the coronary sinus (CS). Subsequently, a circular mapping catheter (LASSO® 2515 Variable Mapping Catheter, Biosense Webster, Irvine, CA, or Inquiry AFII™ Circular Mapping Catheter, 15 or 20 mm, St. Jude Medical, Saint Paul, MN) was advanced to the left atrium via a 12F steerable sheath (8.5F, Agilis NXT; St Jude Medical, Saint Paul, MN) and an ablation catheter with or without contact-force sensing (Smarttouch SF, Biosense Webster, Irvine, CA, or TactiCath or CoolFlex, St. Jude Medical, Saint Paul, MN) was delivered to the left atrium via a non-steerable sheath (SL1, St. Jude Medical, Saint Paul, MN) after double transseptal puncture. 3D mapping was exclusively performed using spiral mapping catheter. Electroanatomical maps with<500 mapping points were excluded from our study group. In principle, cycle length (CL) stability as a mapping criterium was set from −10 ms to + 10 ms of the measured AFL CL. Intracardiac signals recorded by the CS catheter served as reference electrical signal for LA activation mapping. The window of interest was designed to cover at least 90% of the index tachycardia cycle length with the reference electrical CS signal in the middle of the sensing window. Settings for identification of low-voltage areas were set from 0.1 mV to 0.5 mV during electro-anatomical mapping.

RF ablation power was between 25 and 35 W, temperature limit was at 43 °C, and saline irrigation rate at 17–30 mL/min. During ablation at the posterior wall and in the CS, RF power was restricted to 25 W. In both study groups, continuous lines connecting two non-conducting anatomical landmarks within the re-entry were applied. In contrast to the ABA approach, patients in the SBA group were treated by applying line of block taking the individualized LVA pattern into account. Successful ablation was defined as termination of arrhythmia and confirmation bidirectional conduction block. If necessary, additional ablations for pulmonary vein isolation (PVI) or ablation of cavotricuspid isthmus (CTI), were performed.

### 2.3 Postprocedural management and clinical Follow-up

Echocardiography was performed in every patient immediately after the procedure and before hospital discharge to rule out pericardial tamponade or pericardial effusion. Oral anticoagulation was resumed on the day of the ablation procedure. Patients were scheduled for outpatient clinic visits including clinical assessment, echocardiography, 12-lead ECG, and 7-day-Holter-monitoring or 24 h-Holter-monitoring in case of patient’s refusal for longer monitoring at 1, 3 and 6 months after the procedure and thereafter every 6 months.

Any documented sustained atrial arrhythmia on 12-lead rest ECG or any atrial arrhythmia (atrial fibrillation, AF and atrial tachycardia, AT) of ≥ 30 s on Holter ECG or treatment with AAD was counted as AT or AF recurrence episode.

### 2.4 Statistical analysis

Significance of differences of numeric values was calculated by *t*-test if normal distribution with equal variance was given. Normal distribution was determined by Shapiro–Wilk test and equal variance by Brown–Forsythe test. Numeric variables that were not normally distributed were analyzed by Mann–Whitney rank sum test. Categorical variables were analyzed by Chi-square test or Fisher’s exact test. Survival analysis was performed by Kaplan-Meier and log-rank test. A p-value < 0.05 was considered significant. Statistical assessment was performed with Excel (Version 2016, Microsoft Inc., Redmond, WA), XLStat software (V 2016.02.28430, Addinsoft, New York, NY) and SPSS Statistics 25 software (Version 2017, IBM, Armonk, NY, USA).

## 3 Results

### 3.1 Study population

Between February 2012 and February 2018, 60 patients were treated at Ulm University Medical Center for left atrial AFL by a 3D-Mapping and irrigated-tip ablation. 47 of 60 of these patients (78.3%) met the inclusion criteria of our study, while 13 of 60 patients (21.7%) had to be excluded mainly due to inconsistent or missing procedural and/or outcome data. After evaluating left atrial 3D-maps of AFL in a blinded process, 24 of 47 patients (51.1%) were assigned to the substrate-based ablation group (SBA group), whereas 23 of 47 Patients (48.9%) were assigned to the standard anatomical-based ablation group (ABA group).

### 3.2 Baseline characteristics

At the time of enrolment, mean age in the SBA group was 70.0 ± 8.6 years and in the ABA group 69.4 ± 9.9 years (p = 0.94). Most common comorbidities in both study groups were atrial fibrillation (SBA: 87.5%, ABA: 95.7%, p = 0.61) and arterial hypertension (SBA group: 87.5%, ABA group 73.9%, p = 0.29). Mean CHA_2_DS_2_-VASc score in both study groups patients was 3.9 ± 1.8 in the SBA group and 3.4 ± 1.5 in the ABA group (p = 0.39). In 17 of 24 patients (70.8%) of the SBA group and 16 of 23 patients (69.6%) of the ABA group PVI had been performed prior AFL ablation (p = 0.92). Further baseline characteristics associated with the incidence of AFL, such as prior cardiac surgery, mitral valve regurgitation (MVR) or left atrial diameter did also not differ significantly between the SBA and the ABA group (Table 1).

**Table 1:** Baseline characteristics.

Baseline characteristics	SBA group (n = 24)	ABA group (n = 23)	p-value
Age *[y]* [mean ± SD]	70.0 ± 8.6	69.4 ± 9.9	0.94
Sex (female)[n (%)]	7 (29.2)	10 (43.5)	0.31
Body-mass index *[kg/m^2^]* [mean ± SD]	27.6 ± 4.7	28.3 ± 3.8	0.32
Systolic heart failure^1^ [n (%)]	2 (8.4)	2 (8.7)	1.00
Left atrial diameter *[mm]* [mean ± SD]	47.5 ± 6.3	49.1 ± 7.1	0.45
Hypertension [n (%)]	21 (87.5)	17 (73.9)	0.29
Diabetes mellitus [n (%)]	4 (16.7)	0 (0)	0.11
Stroke or TIA [n (%)]	5 (20.8)	3 (13.0)	0.70
Myocardial infaction [n (%)]	3 (12.5)	0 (0)	0.23
Coronary artery disease [n (%)]	9 (37.5)	8 (34.8)	0.85
CHA_2_DS_2_-VASc-Score [mean ± SD]	3.9 ± 1.8	3.4 ± 1.5	0.39
Atrial fibrillation [n (%)]	21 (87.5)	22 (95.7)	0.61
Prior pulmonary vein isolation [n (%)]	17 (70.8)	16 (69.6)	0.92
Prior cardiac surgery [n (%)]	0 (0)	1 (4.3)	0.49
Mitral valve regurgitation [n (%)]	4 (16.7)	2 (8.7)	0.67

1 = defined as left ventricular ejection fraction < 35%.

### 3.3 Procedural data

As shown in Table 2, mean ablation duration was almost identical in both study groups (SBA: 273 ± 101 min vs. ABA: 272 ± 114 min, p = 0.77). An average of 43 ± 34 ablation points were applied in the SBA group, while mean number of 49 ± 45 ablation points were applied in the ABA group (p = 0.67). The average total ablation time of 38 ± 26 min was numerically shorter in the SBA group, compared to 48 ± 45 min in the ABA group. Similar to the total ablation time, fluoroscopy time in the SBA group was also numerically shorter compared to the ABA group (SBA: 32 ± 14 min vs. ABA: 39 ± 22 min). However, despite these numerical differences, ablation duration (p = 0.86) as well as fluoroscopy time (p = 0.19) were statistically not different between both study groups.

**Table 2:** General procedural parameters.

General procedural parameters	SBA group n = 24	ABA group n = 23	p-value
Procedure time (total) *[*min*]* [mean ± SD]	272.9 ± 100.6	272.1 ± 113.8	0.77
Ablation points (total) [mean ± SD]	42.5 ± 34.0	49.4 ± 45.2	0.67
RF time (total) *[*min*]* [mean ± SD]	38.9 ± 25.7	47.8 ± 44.7	0.86
Fluoroscopy time (total) *[*min*]* [mean ± SD]	31.9 ± 14.0	39.3 ± 21.5	0.19

### 3.4 Arrhythmia mechanism and left atrial substrate pattern

In our study population, a total of 60 left atrial AFL in 47 patients were elucidated by 3D mapping. Perimitral flutter as index tachycardia was found in 32/47 patients (68.1%), while roof dependent AFL was found in 15/47 patients at the beginning of the procedure (31.9%). In 13/47 patients (27.7%) additional left atrial macro-reentry tachycardia occurred after successful ablation of the index AFL (6 perimitral and 7 roof-dependent AFL). Additional perimitral flutter appeared in 6 patients after successful ablation of roof-dependent index tachycardia, while secondary roof-dependent atrial flutter patients appeared in 7 patients after successful ablation of perimitral index tachycardia. Mean cycle length was 267 ± 42 ms for perimitral atrial flutter and 284 ± 63 ms for roof-dependent atrial flutter.

Evaluation of the left atrial substrate pattern, derived from the left atrial voltage map during index tachycardia prior ablation, showed low-voltage areas of ≥ 50% of the left atrial myocardium in 40/47 patients of included patients (85%). Overall, there were no statistical differences between the SBA and the ABA group regarding AFL mechanisms, cycle length and left atrial substrate pattern (Table 3).

**Table 3:** Specific procedural parameters.

Specific procedural parameters	SBA group (n = 24)	ABA group (n = 23)	p-value
Index AFL [n (%)]	24 (1 0 0)	23 (1 0 0)	
Perimitral [n (%)]	15 (62.5)	17 (73.9)	0.53
Cycle length *[ms]* [mean ± SD]	270 ± 46	264 ± 37	0.59
Successfully ablated [n (%)]	14 (93.3)	15 (88.2)	1.00
Roof-dependent [n (%)]	9 (37.5)	6 (26.1)	0.53
Cycle length *[ms]* [mean ± SD]	288 ± 83	279 ± 43	0.64
Successfully ablated [n (%)]	8 (88.9)	6 (1 0 0)	1.00
Additional AFL	5 (1 0 0)	8 (1 0 0)	
Perimitral [n (%)]	2 (40)	4 (50)	1.00
Cycle length *[ms]* [mean ± SD]	363 ± 11	270 ± 66	0.16
Successfully ablated [n (%)]	2 (1 0 0)	3 (75.0)	1.00
Roof-dependent [n (%)]	3 (60)	4 (50)	1.00
Cycle length *[ms]* [mean ± SD]	247 ± 37	265 ± 61	0.72
Successfully ablated [n (%)]	3 (1 0 0)	3 (75.0)	1.00
Left-atrial substrate burden^1^			
< 25% [n (%)]	1 (4.2)	0 (0.0)	0.96
25 – 50% [n (%)]	2 (8.3)	4 (17.4)	
50 – 75% [n (%)]	18 (75.0)	15 (65.2)	
> 75% [n (%)]	3 (12.5)	4 (17.4)	
Additional non-AFL ablation			
CTI ablation [n (%)]	3 (12.5)	3 (13.0)	1.00
PVI [n (%)]	4 (16.7)	4 (17.4)	1.00
RePVI [n (%)]	9 (37.5)	10 (43.5)	0.77
FAT ablation [n (%)]	2 (8.3)	5 (21.7)	0.25

1 defined as area of low-voltage ≤ 0.1 mV during AFL mapping.

### 3.5 Distribution of ablation lines in the SBA and in the ABA group

In both study groups, 90.0% (54 out of 60) of the mapped AFL were terminated successfully by ablation. Acute success rate defined as AFL termination during ablation in patients treated with our substrate-based ablation approach was comparable to patients that were treated by the established anatomical-based ablation approach (SBA: 27/29 AFL (93.1%) vs. ABA: 27/31 AFL (87.1%), p = 0.74). Bidirectional line of block was achieved in all patients, in which AFL was successfully terminated by catheter ablation in both the SBA and ABA group. As shown in Fig. 3, ablation lesions in the SBA group were predominantly applied, according to the pattern of LA low-voltage areas, at the LA anterior wall and at the LA roof.

Remarkably, classical mitral isthmus line for the treatment of perimitral AFL was applied in the SBA group for only one AFL, whereas anteroseptal line from the RSPV to the mitral valve (MV), was applied for the treatment of 12 perimitral AFL. Four perimitral AFL in the SBA group were treated by an anterior mitral line from the LSPV to the MV (Fig. 3A).

In case of roof-dependent AFL, 7 AFL in the SBA group were treated with a classical roof-line according to the area of most pronounced scar. Two roof-dependent AFL were terminated by setting ablation lesions from the RSPV to the LSPV including low-voltage areas of the posterior wall and for one AFL including low-voltage areas of the anterior wall. Interestingly, two roof-dependent AFL around the right-sided PV were successfully treated in the SBA group by applying an anteroseptal mitral line reaching from the RSPV to the MV (Fig. 3B).

In the ABA group, 7 perimitral AFL were terminated by a conventional mitral isthmus line from the LIPV to the MV irrespective of left atrial substrate pattern. Four perimitral AFL were treated by applying a lateral mitral line from the LSPV to the MV posterior of the left atrial appendage (LAA). An anterior ablation from the LSPV to the MV anterior of the LAA was performed in five AFL and an anteroseptal line from the RSPV to the MV in five AFL (Fig. 3C). In contrast to the SBA group, roof-dependent AFL (n = 10) in the ABA group was exclusively treated by a conventional roof line (Fig. 3D).

### 3.6 Periprocedural complications

Periprocedural complications occurred in 3/47 patients (6.4%) of the whole study population (supplementary table 1). During ablation procedure, in one ABA patient pericardial effusion with consecutive pericardial tamponade was detected and successfully treated by pericardiocentesis. Further pericardial effusions during or after the procedure were not registered neither in the ABA nor in the SBA group. Further periprocedural complications are presented in supplementary table 1. No other undesirable periprocedural events, in particular neurological complications, vascular complications or sedation-associated complications could be identified in any patient.

### 3.7 Clinical outcome

**Fig. 2:**
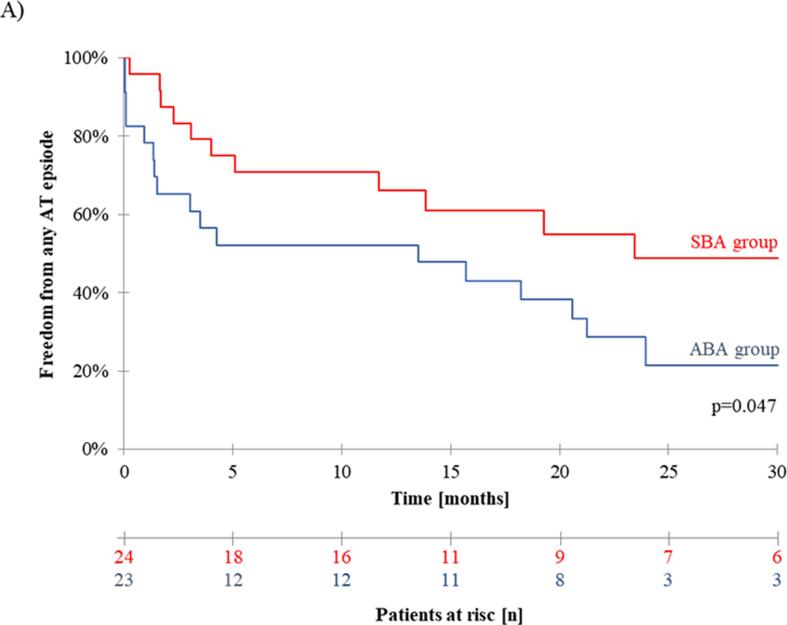
Kaplan-Meier-Survival-Curve: One year after index procedure freedom from AT recurrence of AAD was 66.1% in the SBA group compared to 42.2% in the ABA group (p = 0.047).

After a mean follow-up of 3.0 ± 1.8 years beyond AFL ablation, the clinical endpoint of recurrence of any regular atrial tachycardia (AT), documented by 12-lead or Holter ECG, was met by 12/24 (50%) patients in the SBA group and in 18/23 (78.3%) patients of the ABA group. Remarkably, Kaplan-Meier estimated rate of AT recurrence 12 months after the ablation in the SBA group with 33.9% was significantly lower compared to AT recurrence rate 12 months after ablation in the ABA group (47.8%, p = 0.047, Fig. 2A). During follow-up, drug therapy with either class I or class III antiarrhythmic drugs (AAD) was prescribed in 3/24 SBA patients compared to 7/23 ABA patients (p = 0.32). All AAD treated patients had experienced AT/AF recurrence before AAD prescription.

**Fig. 3:**
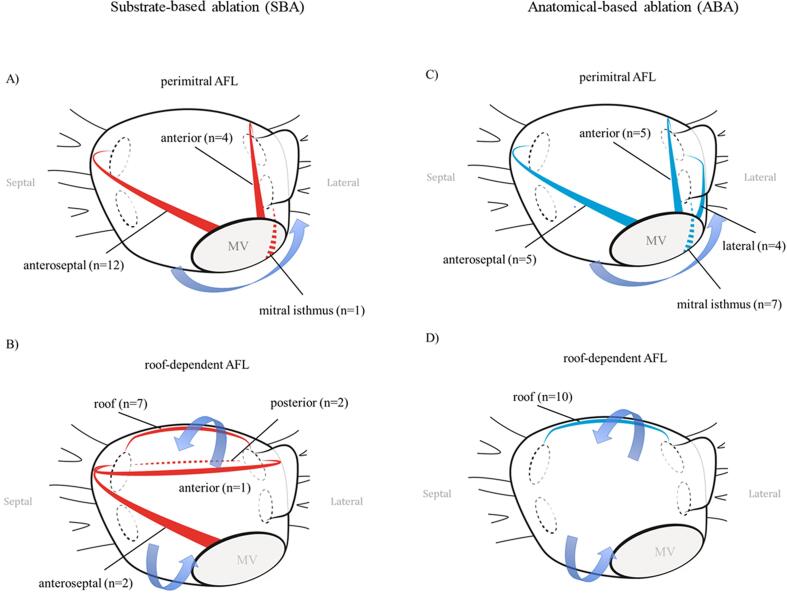
Ablations lines applied in the substrate-based ablation (SBA) group in (A) perimitral atypical flutter (AFL) and in (B) roof-dependent AFL. Application of anatomical based ablation (ABA) lines in (C) perimitral AFL and in (D) roof-dependent AFL.

## 4 Discussion

The present study investigated the use of a novel ablation strategy for catheter ablation of left atrial AFL by incorporating individualized atrial substrate pattern, indicated by low-voltage mapping information, for establishing a line of block within the re-entry circuit.

In our study cohort, both SBA and ABA patients suffer from multiple comorbidities resulting in a high CHA_2_DS_2_-VaSc-Score. Some of these conditions, especially mitral valve regurgitation, arterial hypertension or increased left atrial diameter are clearly associated with LA remodelling resulting in replacement of vital myocardium by fibrotic tissue, also referred as left atrial substrate.

Accordingly, we found that most of the patients in both study groups had advanced LA substrate as indicated by areas of low voltage. Whether endocardial voltage mapping truly detects left atrial substrate is still under debate and especially whether voltage signals detected during atrial tachycardia also represent fibrotic areas in the same way than in sinus rhythm is controversial [14], [15], [16]. However, we found that in comparison to the conventional ablation strategy, procedural success rate in the study group was statistically not inferior. Hence, one might conclude that our strategy targeting low-voltage areas during atrial tachycardia identifies crucial left atrial areas for the sustainment of AFL irrespective from the fact whether endocardial AT voltage-mapping reflects atrial fibrosis in the same way than in SR.

Next to acute procedural success rate, further essential procedural parameters such as complication rates, procedure duration, fluoroscopy and RF ablation time in the SBA group were also similar compared to the established ABA strategy. Especially, reaching the procedural endpoint of a bidirectional line of block within the re-entry circuit was as often achieved in the SBA and ABA group, indicating that incorporating areas of low-voltage in the ablation line is feasible.

Several previous case-series have also evaluated the use of voltage-mapping during AFL for the decision where to apply LA ablation lesions. Unfortunately, either only patients with perimitral AFL were analysed or ablation strategy was restricted to areas of preserved voltage within areas of slow conduction during left atrial AFL without creating a complete line of block. Furthermore, in these studies a control group is missing, limiting interpretation of procedural parameters as well as clinical outcome. [9], [10], [17].

Finally, this is the first study, demonstrating that a substrate-based ablation strategy leads to less AT recurrence rate compared to the established anatomical-based ablation approach. Since AFL requires myocardial substrate for tachycardia sustainment, lower recurrences rates in the SBA group might be explained by reducing left atrial substrate burden in comparison to the ABA group, in which ablation lines were applied irrespective of low-voltage areas. Alternatively, higher recurrence rate in the ABA group, in which healthy tissue was ablated, might be caused by line re-connection compared to the SBA group, which is an important mechanism of AT recurrence.

To summarize, our study suggests that a substrate-based ablation strategy is a feasible, effective and safe approach for the treatment of left atrial macro-reentry tachycardia and leads to less arrhythmia recurrences compared to the conventional anatomical based ablation.

## 5 Limitations

The present study contains several shortcomings that might limit the scientific quality and translation to daily clinical practice. First, data were collected only from one EP center and this study is of retrospective nature. Furthermore, the study cohort is rather small and mainly older patients, which suffered also from atrial fibrillation and had signs of atrial cardiomyopathy, were included. Hence, one might speculate whether findings of this selected patient group are transferable to younger AFL patients without signs of atrial cardiomyopathy. AFL Mapping was performed using standard spiral mapping catheter allowing to elucidate all macro-reentries in our study cohort and identifying areas of low voltage in the left atrium. Remarkably, we found no post-PVI related AFL, which might be either a finding by chance or might reflect the fact that mapping was not performed by high-density (HD) mapping catheters.

## Funding

C. Schweizer and Y. Teumer were funded by the Deutsche Herzstiftung. The other authors have no conflicts to report.

## Ethical approval

This study was approved by our local Ethic Committee of the University Ulm/Germany (Ref-Number: 324/16).

## 8 Authoŕs contribution

A. Pott: Study design, data collection, data analysis, interpretation, and manuscript compilation.

Y. Teumer: Data collection, data analysis, interpretation, critical review of the manuscript.

K. Weinmann, M. Baumhardt, C. Schweizer, D. Buckert, C. Bothner: data collection and analysis, critical review of the manuscript.

W. Rottbauer: critical review of the manuscript.

T. Dahme: Study design, data collection, data analysis, interpretation, and manuscript compilation.

## Declaration of Competing Interest

The authors declare that they have no known competing financial interests or personal relationships that could have appeared to influence the work reported in this paper: [T. Dahme received speaker’s honoraria and consulting fees from Medtronic, Biosense Webster, Boerhringer-Ingelheim, Bayer, Daiichi-Sankyo. A. Pott received speaker’s honoraria from Medtronic, Biosense Webster, Daiichi-Sankyo and is invited fellow of the Boston Scientific EP training program.].
