# Supplementary material for: Substrate-based ablation of atypical atrial flutter in patients with atrial cardiomyopathy
Source: Int J Cardiol Heart Vasc. 2022 Apr 18;40:101018. doi: 10.1016/j.ijcha.2022.101018 (PMC9043977; doi:10.1016/j.ijcha.2022.101018)
Supplement: Supplementary data 1 [file mmc1.docx]

**Supplements:**

Supplementary table 1:

| Periprocedural complications | SBA group (n=24) | ABA group (n=23) | p-value |
| --- | --- | --- | --- |
| Pericardial effusion [n (%)]  Pericardial tamponade [n (%)]  Neurological complications [n (%)]  Stroke [n (%)]  TIA [n (%)]  Cranial bleeding [n (%)]  Clinical apparent bleeding [n (%)]  Vascular complications [n (%)]  Sedation associated complications [n (%)] | 0 (0)  0 (0)  0 (0)  0 (0)  0 (0)  0 (0)  1 (4.2)  0 (0)  0 (0) | 1 (4.3)  1 (4.3)  0 (0)  0 (0)  0 (0)  0 (0)  1 (4.3)  0 (0)  0 (0) | 0.59 |
